# Exploring Divergent Views: A Comparative Study of Uterus Transplantation Perceptions among Transplant and Obstetrics/Gynecology Providers

**DOI:** 10.3390/jcm13113182

**Published:** 2024-05-29

**Authors:** Prema Vyas, Danielle Sader, Giuliano Testa, Jinyu Du, Anji Wall, Liza Johannesson

**Affiliations:** 1 Burnett School of Medicine, Texas Christian University, Fort Worth, TX 76109, USA; 2 Department of Surgery, Baylor Annette C. and Harold C. Simmons Transplant Institute, Baylor University Medical Center, Dallas, TX 75246, USA; giuliano.testa@bswhealth.org (G.T.); jinyu.du@bswhealth.org (J.D.); anji.wall@bswhealth.org (A.W.); 3 Department of Obstetrics and Gynecology, Baylor University Medical Center, Dallas, TX 75246, USA

**Keywords:** uterus transplantation, ethics, absolute uterine-factor infertility, survey

## Abstract

**Background**: Uterus transplantation (UTx) provides women with absolute uterine-factor infertility (AUFI) the opportunity to carry their own pregnancy and deliver a child. There are multiple ethical and medical concerns associated with UTx. Since the last survey of US provider perceptions of UTx in 2018, there have been additional reports of successful transplantations and pregnancies. This study aimed to identify the perception of UTx among providers involved in the diagnosis of AUFI and on the transplant team to help us understand knowledge gaps and determine what barriers must be overcome for UTx to be used in general clinical practice. **Methods**: We administered REDCap surveys to conference attendees at the 2023 American College of Obstetricians and Gynecologists (ACOG) conference and 2023 American Transplant Congress (ATC). Participants were recruited by medical student volunteers. **Results**: Two hundred ACOG and ATC attendees completed the survey. Medical concerns related to UTx were reported by 42% of providers from ACOG compared to 22% of providers from ATC. Overall, 76% of participants agreed that UTx should be an option for patients with congenital AUFI. Lastly, 68% of participants agreed that the procedure should be presented as an option for transgender women. **Conclusions**: This study further elucidates the perception of UTx among obstetricians/gynecologists and transplant physicians. We found greater support for the procedure than in previous studies. This study also demonstrates provider support for presenting this procedure as an option for transgender women.

## 1. Introduction

Uterus transplantation (UTx) is a successful treatment for women with absolute uterine-factor infertility (AUFI) who desire to experience pregnancy and childbirth. The first UTx in the United States was performed in 2016, and the first live birth occurred 1 year later in 2017 [1]. To date, more than close to 50 UTx have been performed in the country with 35 babies born [2].

Due to the rapid expansion of UTx, providers who interact with patients who have AUFI may have a gap in knowledge about UTx, which may influence their opinions of this procedure. Only a few published reports have specifically examined the opinions and perceptions of healthcare providers on UTx. An interview study of UK healthcare professionals in 2015 investigated perceptions toward UTx [3]. This study found that out of 528 participants, 94% thought that UTx is a viable option for patients with AUFI as long as it meets the appropriate medical and ethical criteria. Although providers were supportive, only 57% believed that the procedure would be achievable in clinical practice [3]. A 2018 survey of physician members of the American Society of Reproductive Medicine (ASRM) and the American Association of Gynecologic Laparoscopists (AAGL), mostly consisting of reproductive endocrinologists and minimally invasive surgeons, reported that 90% of respondents would not recommend UTx to their patients and that only 28% believed that the recipients’ risk was acceptable [4]. Two years later, in 2020, another interview study of US obstetricians, gynecologists, transplant surgeons, internists, and family practitioners at three Mayo Clinic sites found that most respondents might (46%) or would (36%) introduce the option of UTx to AUFI patients [5]. However, this study had a low response rate (23%), and only 80 of the 447 respondents (18%) stated that UTx was relevant to their practice. In addition, in 2018, the ASRM released a position statement that cautioned healthcare providers and the public on the highly experimental and novel nature of UTx [6]. Of note, this position statement as well as these surveys were conducted during the early experimental phase of UTx. 

Given the advancement of UTx and its continued clinical success since the conclusion of previous survey studies, it is important to re-evaluate the perceptions and opinions of providers who are involved in the diagnosis and treatment of patients with AUFI. To achieve this aim, we developed a survey for transplant and obstetricians/gynecologists (ob/gyns) aimed at identifying current perceptions and concerns about UTx. 

## 2. Materials and Methods

### 2.1. Survey Development 

A survey instrument was designed in REDCap 14.0.01 (Reseach Electronic Data Capture) based on previously published surveys developed to assess perception of UTx [4,5]. The survey included a brief paragraph providing a basic introduction to UTx, seven demographic questions, and nine statements about UTx with Likert-scale answer choices (Appendix A). Biostatisticians and principal investigators were asked to review the survey for content and data collection. 

### 2.2. Survey Distribution 

The survey was distributed to medical providers attending either the American College of Obstetricians and Gynecologists (ACOG) Annual Clinical and Scientific Meeting in Baltimore, Maryland, 19–21 May 2023, or the American Transplant Congress (ATC) Meeting in San Diego, California, 3–7 June 2023. Conference attendees were approached at random by two investigators (DS and PV) and asked if they were willing to participate in the survey. Responses were collected by the investigator on a tablet or a smartphone. Participants also had the option to scan a QR code that allowed them to complete the survey on their own mobile or tablet device. Attendees were excluded from completing the survey if they were not clinical providers. 

### 2.3. Data Analysis 

Descriptive demographic information (age, gender, race/ethnicity, current role, work setting, specialty, years of practice in the current role) stratified by conference groups (ACOG, ATC) was collected. Fisher’s exact test for homogeneity was performed to test differences in the demographic characteristics. Stacked bar charts were made to analyze responses for the nine Likert-scale questions. A Kruskal–Wallis test was performed to test for differences in opinion tendencies of survey respondents based on conference, age, gender, and race/ethnicity. A *p* value < 0.05 was considered significant. All data analysis was performed using RStudio version 4.3.2. 

## 3. Results

### 3.1. Demographic Data 

A total of 200 conference attendees participated in this study. The participants were equally distributed between the two conferences (102 at ACOG and 98 at ATC). Table 1 shows the demographic information of survey respondents stratified by conference groups. ACOG respondents were younger (41.2% were 25–35 years old) than ATC respondents (21.4% were 25–35 years old). The number of female respondents (81/79.4%) was four times that of male respondents (21/19.6%) for ACOG, whereas there were about equal numbers of female and male respondents for ATC. For both conference groups, most respondents were Non-Hispanic White/Caucasian (40.2% for ACOG and 53.1% for ATC). ACOG had a larger proportion of Non-Hispanic Black or African American respondents (29.4%) than ATC (10.2%). The proportion of Asian respondents for ACOG and ATC was 19.6% and 26.5%, respectively. In terms of the current role, most respondents were attending physicians (57.8% for ACOG and 67.3% for ATC). Regarding specialty, 70.6% of the ACOG respondents were general obstetricians/gynecologists, while 26.5% of ATC respondents were transplant physicians and 22.4% were transplant surgeons. In terms of experience, 41.2% of respondents at ACOG and 22.4% at ATC reported <5 years of practice; 14.3% of respondents at ATC had 25+ years of practice vs. 2% for ACOG. Fisher’s exact test for homogeneity showed that the two conference groups (ACOG, ATC) had statistically significant differences (*p* < 0.05) in the distribution of age group, gender, race/ethnicity, work setting, specialty, and years of practice in the current role.

### 3.2. Perception of Uterus Transplantation 

#### 3.2.1. Relevance to Practice

**Figure 1:**
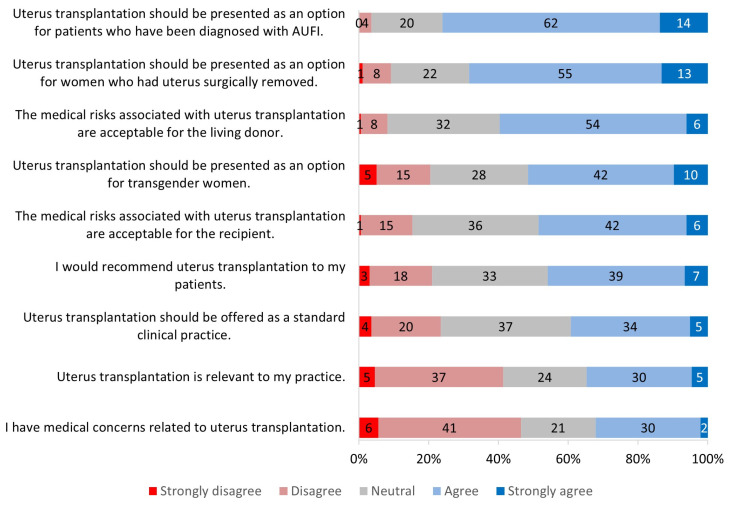
Overall survey results.

**Figure 2:**
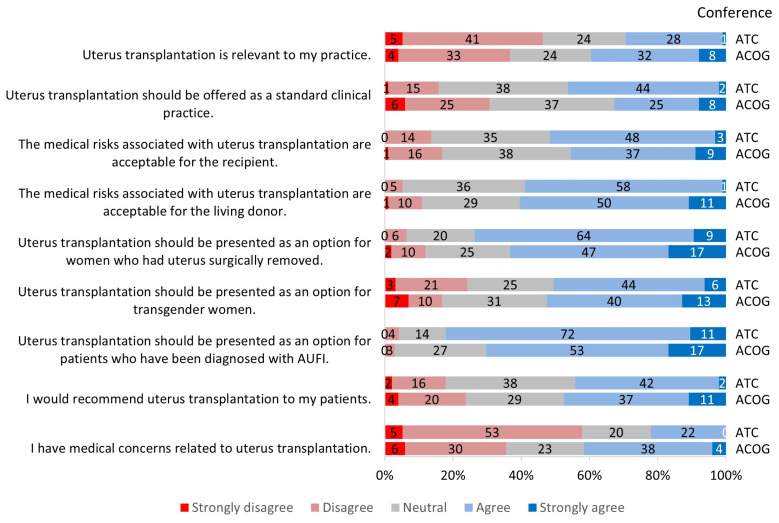
Survey responses by conference groups.

**Figure 3:**
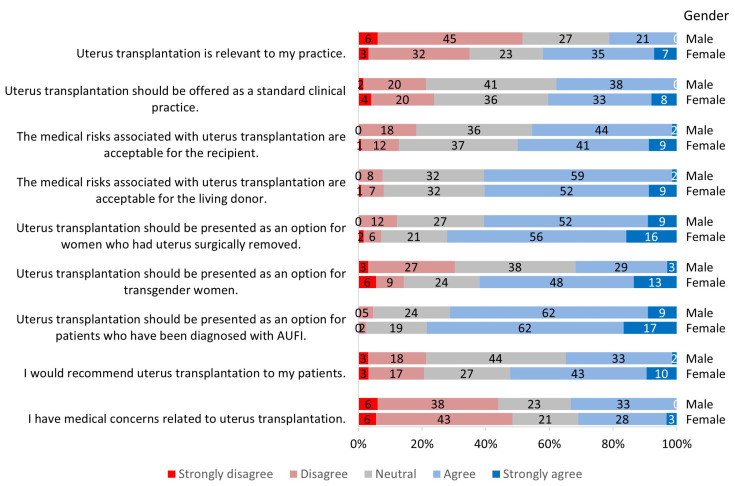
Survey responses by gender groups.

**Figure 4:**
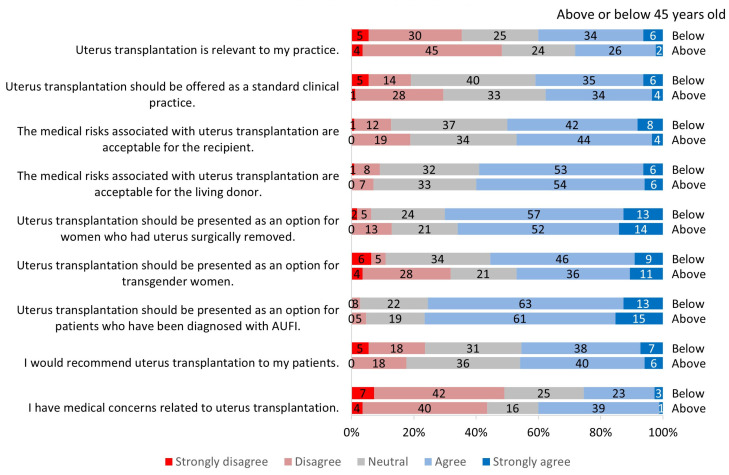
Survey responses by age groups.

When asked about the relevance of UTx, 35% of all participants agreed or strongly agreed that it was relevant to their practice (Figure 1); 40% of ACOG respondents responded this way compared to 29% of ATC respondents (Figure 2). When comparing gender groups, female participants reported a significantly higher relevance (*p* = 0.003) to their practice than male participants (Figure 3). The gender difference was regardless of conference participation and medical specialty. When comparing age groups, 48% of providers ≥45 years of age found this procedure to be more relevant to their practice compared with 35% of those <45 years, but this was not statistically significant (Figure 4).

#### 3.2.2. Clinical Candidacy for Uterus Transplantation

Participants were queried concerning potential patient populations for UTx. Overall, 46% would recommend UTx to their own patients (Figure 1). There was a significant difference between male and female participants, where female participants reported a higher willingness to recommend that their patients undergo UTx (52% vs. 35%) (*p* = 0.04) (Figure 3). The gender difference was regardless of conference participation and medical specialty.

Participants were also asked about whom UTx should be offered to. These options included patients who had been diagnosed with congenital AUFI, transgender women, and women who have had their uterus surgically removed. While 76% of participants agreed that UTx should be an option for patients with congenital AUFI (Figure 1), 68% of participants agreed that the procedure should be presented as an option for transgender women. Gender differences were evident for this question: 62% of female participants agreed that UTx should be an option for transgender women versus 32% of male participants (*p* < 0.001) (Figure 3). Lastly, for patients who had their uterus surgically removed, 68% of participants agreed that UTx should be presented as an option (Figure 1). There was no significant difference in responses based on participant specialty, gender, or ethnicity.

#### 3.2.3. Risks Associated with Uterus Transplantation 

Perceptions of risks were evaluated by asking about the acceptable medical risks associated with UTx for the living donor and the recipient. Overall, 60% of participants agreed that the risks for the living donor were acceptable, while 48% of the participants believed that the risk was acceptable for the recipient (Figure 1). Perception of risks were also assessed by asking them to respond to the statement, “I have medical concerns related to UTx”. Overall, 46% of participants disagreed, 21% were neutral, and 32% agreed with the statement (Figure 1). 

**Figure 5:**
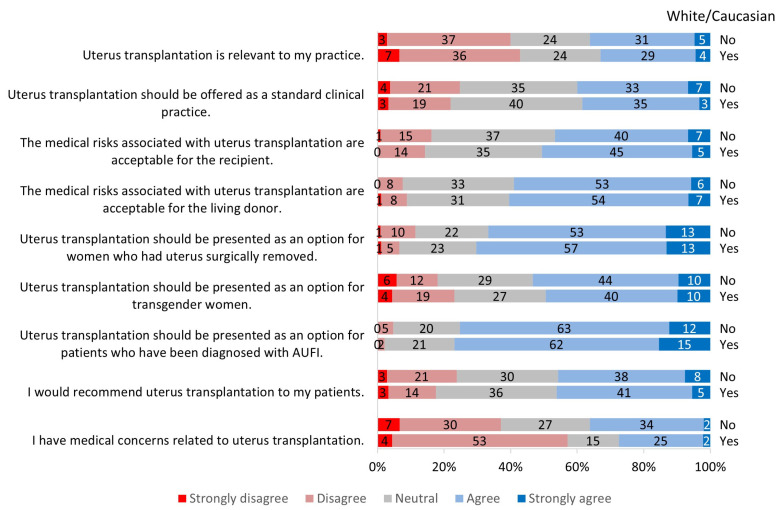
Survey response by race/ethnicity groups.

There was a significant difference between conference groups related to the statement “I have medical concerns related to UTx” (*p* = 0.0015): 42% of participants from ACOG responded that they had medical concerns related to UTx compared to 22% of the providers at ATC (Figure 2). In addition, there was a significant difference between White/Caucasian and Non-White/Non-Caucasian groups: 36% of the Non-White/Non-Caucasian group agreed to having medical concerns, while only 27% of participants in the White/Caucasian group expressed medical concerns (*p* = 0.04) (Figure 5). The other statements used to assess risk did not show any significant differences between the various groups. 

#### 3.2.4. Future of Uterus Transplantation 

The last statement that participants were asked to respond to was, “UTx should be offered as a standard clinical practice”. Overall, 39% of all participants believed that the procedure should be a standard medical procedure offered to patients (Figure 1); 37% felt neutral toward the statement, and 23% of participants disagreed. There was a significant difference in opinions between respondents at ACOG and ATC (*p* = 0.02): 46% of respondents at ATC agreed that it should be standard clinical practice, while only 33% of respondents at ACOG agreed (Figure 2). In addition, 37% and 31% of ACOG providers reported that they felt neutral or disagreed, respectively (Figure 2).

## 4. Discussion

The current study was conducted to determine perceptions of UTx in the transplant and ob/gyn medical communities. We found that more ACOG participants believe that UTx is relevant to their practice than transplant providers (40% vs. 29%). This is similar to the results of a previous report from 2020, where almost half of ob/gyns and one third of the transplant providers agreed that UTx was relevant to their practice (15). While only one third of all participants (32%) in the current study believe that UTx is relevant to their own practice, close to half of all participants (46%, and 48% of ob/gyn providers) would recommend UTx to their patients. This is higher than the 2017 study of members of AAGL and ASRM, where only 11% stated they would recommend UTx as a treatment to their own patients [4]. The increasing willingness to recommend UTx may be due to increased recognition that UTx is a viable clinical option for women with AUFI. 

To date, UTx has been performed only in women with either a congenitally absent uterus or a surgically removed uterus (due to myomas, previous cancer treatment, obstetrical bleeding, etc.). Two previous studies [4,5] have reported support for UTx in women with severe AUFI, which was defined as “congenital absence of the uterus” (42% [4] and 36% [5], respectively). Our results demonstrate a higher percentage (76%) of overall support for the procedure to be performed in patients with unspecified AUFI, which includes surgically induced AUFI. This increase in support further strengthens the idea that providers are possibly more comfortable with the procedure and willing to recommend it to patients with unspecified AUFI. 

The increasing success of UTx in cis women has triggered interest in extending application of the procedure to other populations, such as transgender women [7]. In a British study from 2015, transgender women were surveyed on their perception of UTx and reported that the transplant may improve their quality of life and help with symptoms of gender dysphoria [7]. When we asked specifically if UTx should be suggested for transgender patients, the majority of respondents (68%) agreed. This is a higher percentage than in the 2020 report from the Mayo Clinic, in which only 18% of respondents believed that transgender women would be good candidates for the procedure [5]. Given the increasing support, these results may provide UTx programs with the confidence to offer the procedure to transgender patients. However, the impact of expanding the scope of UTx needs further evaluation because it may impact perceptions of acceptability of the procedure in general.

A report of the first 5 years of clinical experience of UTx in the US shows that complications regardless of severity following UTx are a concern not only for the recipients but also for the living donor and the potential child [2]. The ethical concerns as well as the risks and benefits for the donor, recipient, and children related to the surgeries involved, the immunosuppressive treatment, the pregnancy, and the delivery are not fully known and have been vigorously debated [2]. Our survey results show that 60% of providers agreed that the risk for living donors is acceptable, while only 48% found the risk of UTx for recipients is acceptable. This supports the findings of Bortoletto et al. where 54% of providers reported being comfortable with the risks for living donors but only 28% were comfortable with the risks to the recipient [4]. The lower concern for donor risk could be explained by the relative novelty of UTx versus more familiarity with a hysterectomy procedure, albeit a donor hysterectomy is more complex than a simple hysterectomy. 

This additional concern for the recipient may be explained by the partly unidentified risk of a planned pregnancy and immunosuppressive treatment given to the recipient for the duration of the graft-recipient time (from UTx to graft hysterectomy following childbirth). There has been an increased rate of solid organ transplantation among reproductive-aged patients [8]. Currently, immunosuppression management in UTx derives from protocols from other solid organ immunosuppression, most commonly the kidney. While the risk of pregnancy and immunosuppression is well known in other solid organ recipients, mostly kidney transplant recipients, many have posed the argument that patients undergoing UTx are healthier with far fewer comorbidities than patients undergoing other solid organ transplants; therefore, they anticipate fewer obstetric and immunosuppressive complications [9]. In addition, UTx recipients are immunocompromised for a shorter period compared to other transplant recipients. We anticipate that as more long-term studies focus on the effects of immunosuppressants on the recipient and on fetal well-being, this will help elucidate the medical risks related to the recipient. 

Two US programs, the Baylor University Medical Center, Dallas, and the University of Alabama, Birmingham, offer UTx as standard clinical practice outside of a clinical trial. Our survey results found that a higher proportion of transplant providers than ob/gyn providers agreed that UTx should be standard clinical practice (46% and 33%, respectively). These results suggest that despite the increase in utilization of UTx and increasing clinical success, the majority of providers still believe that UTx is a research procedure. Further research is needed to determine what physicians need to be comfortable considering UTx a standard clinical procedure (e.g., education, clinical outcomes). Education of ob/gyns about the current state of UTx may be beneficial in increasing the acceptance of UTx as clinical practice in the medical community. Future studies should focus on the reasons that providers do not accept the clinical application of UTx and how these concerns can be addressed. 

There are several limitations to this study. First, the specialty of over 50% of survey respondents from ATC was reported as “other”. This can be explained by the large multidisciplinary approach to transplant medicine that involves pharmacists, nurse practitioners, and other healthcare professionals. In addition, the survey format did not allow us to understand the reason for providers’ beliefs and motivations. Future studies would benefit from interview studies or studies with open-ended responses to allow physicians to provide additional reasoning and motivations. Lastly, due to the nature of survey distribution, we were unable to report a response rate. Given that we had total of 200 participants and all were approached at random, we are not concerned about this lack of information.

Ultimately, this study further elucidates the perception of UTx among ob/gyns and transplant physicians. We found greater support for the procedure among providers compared to previous studies. Future studies should focus on identifying knowledge gaps and misperceptions of UTx to identify areas in which provider education will be most helpful. 

## Figures and Tables

**Table 1:** Demographic information for survey respondents.

		Conferences	
		ACOG	ATC
**Participants (*n*)**		**102**	**98**
**Age**	25–35 years old	42 (41.2%)	21 (21.4%)
	36–45 years old	18 (17.6%)	30 (30.6%)
	46–55 years old	24 (23.5%)	34 (34.7%)
	56–65 years old	16 (15.7%)	11 (11.2%)
	66+ years old	2 (2.0%)	1 (1.0%)
**Gender**	Female	81 (79.4%)	47 (48.0%)
	Male	20 (19.6%)	48 (49.0%)
	Not listed (please specify)	1 (1.0%)	1 (1.0%)
	Prefer not to answer	0 (0.0%)	1 (1.0%)
**Race/ethnicity**	Asian	20 (19.6%)	26 (26.5%)
	Hispanic, Latino, or Spanish origin	9 (8.8%)	5 (5.1%)
	Middle Eastern or North African	0 (0.0%)	3 (3.1%)
	Non-Hispanic Black or African American	30 (29.4%)	10 (10.2%)
	Non-Hispanic White/Caucasian	41 (40.2%)	52 (53.1%)
	Other	2 (2.0%)	2 (2.0%)
**Professional role**	Attending	59 (57.8%)	66 (67.3%)
	Fellow	7 (6.9%)	3 (3.1%)
	Resident	18 (17.6%)	10 (10.2%)
	Other	17 (16.7%)	18 (18.4%)
**Professional** **setting**	Community clinic or hospital	23 (22.5%)	13 (13.3%)
	Military/government	5 (4.9%)	3 (3.1%)
	Multispecialty group or private practice	2 (2.0%)	0 (0.0%)
	Other (please specify)	7 (6.9%)	3 (3.1%)
	Single-specialty group private practice	11 (10.8%)	0 (0.0%)
	Staff model HMO	3 (2.9%)	0 (0.0%)
	University or teaching hospital or medical center	50 (49.0%)	78 (79.6%)
	Other	1 (1.0%)	1 (1.0%)
**Medical specialty**	Complex family planning	4 (3.9%)	0 (0.0%)
	General obstetrician/gynecologist	72 (70.6%)	0 (0.0%)
	Gynecological oncologist	1 (1.0%)	0 (0.0%)
	Maternal-fetal medicine specialist	10 (9.8%)	0 (0.0%)
	Reproductive endocrinologist	3 (2.9%)	0 (0.0%)
	Transplant physician	0 (0.0%)	26 (26.5%)
	Transplant surgeon	0 (0.0%)	22 (22.4%)
	Other (please specify)	12 (11.8%)	50 (51.0%)
**Years of practice**	<5 years	42 (41.2%)	22 (22.4%)
	6–10 years	14 (13.7%)	12 (12.2%)
	9–15 years	7 (6.9%)	17 (17.3%)
	16–20 years	22 (21.6%)	16 (16.3%)
	21–25 years	15 (14.7%)	16 (16.3%)
	>25 years	2 (2.0%)	14 (14.3%)

## Data Availability

The data presented in this study are available on request from the corresponding author.

## Appendix A

### Background Information

Uterus transplantation (UTx) is a proven treatment for women impacted by absolute uterine-factor infertility (AUFI). The temporary graft (removed after childbirth) allows these women who could not be treated previously to carry a pregnancy and provides options to patients who live in areas where surrogacy is prohibited. Prior to UTx, women with AUFI were limited to using gestational carriers or adoption to build a family.

The first transplant in the United States took place in 2016; since then, a group of institutes has formed the United States Uterus Transplant Consortium (USUTC) [2]. From 2016 to 2022, 37 UTx were performed in the US and 27 babies were born [2]. The success rate (delivery of a baby) is currently >80%.

This procedure begins with the surgical removal of the donor uterus, either as an open procedure or as a complete/partial robotic-assisted procedure. The graft can be donated from either a living or deceased donor. Once the organ is transplanted into the recipient, the recipient begins immunosuppression to avoid graft rejection. Clinical signs of graft function are observed when menstrual bleeding starts, which has been reported at a median of 30 days [2]. Pregnancy is achieved through assisted reproductive technology. All deliveries are currently being performed via cesarean section due to concerns of vaginal anastomosis and damage to structures during labor [2]. Presently, a transplanted uterus is only utilized for a maximum of two to three pregnancies [10]. Patients will then undergo a graft hysterectomy in order to discontinue immunosuppressive medications.

**Survey**

**Demographics**

1. **What is your specialty?**

☐ General obstetrician/gynecologist
☐ Maternal fetal medicine specialist
☐ Reproductive endocrinologist
☐ Gynecological oncologist
☐ Pediatric & adolescent gynecologist
☐ Complex family planning
☐ Transplant surgeon
☐ Transplant physician
☐ Other (please specify): ______________

2. **What is your current professional role?**

☐ Resident
☐ Fellow
☐ Attending

3. **Gender:**

☐ Male
☐ Female
☐ Not listed (Please specify)
☐ Prefer not to answer

4. **How old are you?**

☐ 25–35 years old
☐ 36–45 years old
☐ 46–55 years old
☐ 56–65 years old
☐ 66+ years old

5. **Please identify your race/ethnicity:**

☐ Non-Hispanic White/Caucasian
☐ Non-Hispanic Black or African American
☐ Asian
☐ American Indian or Alaska Native
☐ Native Hawaiian or Other Pacific Islander
☐ Middle Eastern or North African
☐ Hispanic, Latino, or Spanish origin
☐ Other (please specify): __________________________

6. **How long have you been practicing as an attending?**

- 0–5 years
- 6–10 years
- 11–15 years
- 16–20
- 21–25 years
- 26+ years

7. **What best describes the medical setting you practice in?**

- University or teaching hospital or medical center
- Community clinic or hospital
- Single- specialty group private practice
- Staff model HMO
- Fertility clinic
- Military/government
- Multispecialty group or private practice
- Other (please specify)_________________________

**Please respond to the following statements:**

	**Strongly** **Disagree**	**Disagree**	**Neutral**	**Agree**	**Strongly** **Agree**
I have medical concerns related to uterus transplantation					
I would recommend uterus transplantation to my patients					
Uterus transplantation should be a viable option for patients who have been diagnosed with congenital AUFI					
Uterus transplantation should be a viable option for transgender women					
Uterus transplantation should be a viable option for women who have had their uterus surgically removed					
The medical risks associated with uterus transplantation are acceptable for the donor					
The medical risks associated with uterus transplantation are acceptable for the recipient					
Uterus transplantation should be a standard clinical practice					
Uterus transplantation is relevant to my practice					
